# Quality and readability of online patient information on treatment for erectile dysfunction

**DOI:** 10.1002/bco2.87

**Published:** 2021-05-06

**Authors:** Trent A. Pattenden, Rachael A. Raleigh, Elle R. Pattenden, Isaac A. Thangasamy

**Affiliations:** ^1^ Department of Urology Ipswich Hospital Ipswich QLD Australia; ^2^ School of Medicine Griffith University QLD Australia; ^3^ Department of Pharmacy Gold Coast Hospital and Health Service Southport QLD Australia; ^4^ Melbourne School of Psychological Sciences Faculty of Medicine, Dentistry and Health Sciences The University of Melbourne Melbourne VIC Australia; ^5^ Faculty of Medicine University of Queensland Herston QLD Australia

**Keywords:** erectile dysfunction, health information, internet, readability, treatment

## Abstract

**Objectives:**

To investigate the quality and readability of online patient information on treatment for erectile dysfunction using a Google search.

**Materials and methods:**

The results of a Google search for “erectile dysfunction treatment” were reviewed. Webpages that contained written information on erectile dysfunction except those containing scientific publications and paywall protected webpages were included in further analysis. Typographic and treatment information were recorded. Readability was assessed using the Fleisch‐Kincaid grade level, the Gunning‐Fog index, the Coleman‐Liau index, and Simple Measure of Gobbledygook. Website quality was assessed using the DISCERN instrument, Journal of the American Medical Association (JAMA) benchmark criteria, and presence of Health on the net (HON) code certification. Website typography, discussed treatment types, readability scores, and quality measures were reported. Parametric and nonparametric statistical tests were used to compare the data as appropriate dependent on the normality of data.

**Results:**

Eighty‐one webpages were included. Urologists and hospitals were the most common producers with 15 (18%) each. Seventy‐four (91%) webpages contained specific information on treatment for erectile dysfunction and 15 (19%) contained advertisements. Seventeen (21%) webpages were HON code certified. The median DISCERN score was 35 (IQR 26.5‐44) out of 80. The mean combined readability score was 12.32 (SD 1.91). The median JAMA benchmark score was 1 (IQR 1‐2) out of 4. Google rank had a small negative correlation with DISCERN score (*τ* = −0.16, *P* = .036). HON code certified webpages had higher DISCERN scores (median of 44 [IQR 35‐58.5] vs 32.5 [IQR 25.25‐42.25], U = 832, Z = 6561, *P* < .001). A linear regression was used to predict DISCERN score based on meeting each JAMA benchmark criterion (F(2, 78) = 22.7, *P* < .001) *R* ^2^ = 0.368, *P* < .001. Within this model the effects of meeting attribution (*β* = 11.09) and currency (*β* = 8.79) criterion were significant.

**Conclusions:**

The quality of online information on treatment for erectile dysfunction is generally poor. However, easy to identify markers of quality like HON code certification, or meeting JAMA benchmark criterion for attribution and currency may help patients to navigate to better quality online information on treatment for erectile dysfunction. Webpages are written at senior high school level, above any recommendations for patient medical information. Health professionals should use validated instruments to assess the quality of online information on treatment for erectile dysfunction prior to publication to improve their utility for patients.

## 1 INTRODUCTION

Erectile dysfunction (ED), defined as the inability to gain or maintain an erection firm enough for sexual activities is a common sexual disorder in men.^1^ The prevalence of ED increases with age; from less than 10% in the fifth decade to more than 30% in the seventh decade of life.^2^ Most men do not seek treatment for their symptoms; for example, in a cohort of Australian men, only 38% sought treatment.^3^

People, however, increasingly search the internet for medical information. A cross‐sectional study of 2944 Australian patients in general practice found 28% of participants searched the internet for medical information in the previous month.^4^ Another Australian study of 400 emergency department patients found 49% regularly searched the internet for medical information, and 35% searched their current issue prior to presenting.^5^ In this cohort, 94% used a Google search to find information.

A certain level of health literacy is required to interpret information on the internet appropriately. In Australia, 59% of people aged 17‐74 years were assessed as having inadequate health literacy necessary for understanding information related to their health issues.^6^ This level is even higher in the elderly population.^7^ Ensuring patient education material is written at an appropriate readability level for the target audience is recommended to improve their utility ^8^. Readability is a measure of “the ease of reading words and sentences”.^9^ In the United States of America the recommended readability level is at or below fifth grade.^9^ A national Australian target does not exist though some state health departments have their own recommendations from a sixth‐ to eighth‐grade level.^10^, ^11^, ^12^, ^13^ Online urological information is often more difficult to read than recommended. A study of online information from academic urology departments in the USA and the AUA reported the average readability grade level of each institution's material ranged from 11.1 to 16.5.^14^

Previous research on online medical information has found many sites are of poor quality, are inaccurate, or incomplete. These undesirable characteristics limit their trustworthiness.^15^ A review of information on urology topics on social media was found much commercial in nature, biased, or misinformative.^16^ One study has looked at the quality of medical information within YouTube videos on ED, finding many videos were of poor quality, and 22% attempted to sell specific treatments to viewers.^17^

This study aims to assess the quality of online information on ED treatment using the search strategy utilized most by patients; a Google search.

## 2 METHODS

### 2.1 Webpage selection

A Google search was conducted for the term “erectile dysfunction treatment” on 24 November 2020. This was conducted using Google Chrome (Version 87.0.4280.67), within a new installation to limit any tracking cookies affecting results. Ignoring the promoted advertisements, the first 100 webpage results were assessed by two reviewers against the inclusion and exclusion criteria. Pages that contained written information on ED were included. Exclusion criteria were webpages containing scientific information presented as peer reviewed journal articles or books, and webpages that were paywall protected. Websites that contained information on multiple pages that both reviewers believed were linked as a single item were reviewed collectively as one webpage.

### 2.2 Demographic and content information

Pages were reviewed and information were extracted. The reviewers recorded pages typology and if advertisements were present, webpages contained specific information about ED treatment, and treatments were specifically described (as opposed to listed without any description).

### 2.3 Readability measures

The readability of each webpage was assessed using four validated measures: Fleisch‐Kincaid Grade Level (FKG), the Gunning‐Fog Index (GFI), Coleman‐Liau Index (CLI), and the Simple Measure of Gobbledygook (SMOG). The online readability score calculator Readable was used to calculate the four readability scores for each page.^18^ This was completed by copying the body text from each page into the score text function.

FKG weighs texts with longer sentence and multisyllabic words as being more difficult to understand.^19^ GFI is similar to FKG, however, it counts complex words (those with three or more syllables) rather than total syllable count.^20^ CLI considers word length by character count rather than syllable count.^21^ SMOG also assesses complexity by counting polysyllabic words, however, it only assesses samples of text; 30 sentences in total, 10 from the start, 10 from the middle, and 10 from the end of the text.^22^ All four measures provide a score which is equivalent to the school grade‐level comprehension required for readability, and have previously been used to assess readability of online patient education material from the European Association of Urology.^23^, ^24^

### 2.4 Quality measures

The quality of each page was assessed using three recognized measures: Health on the Net (HON) Code Certification, the Journal of the American Medical Association (JAMA) benchmark criteria, and DISCERN criteria.

HON code certification was determined using the HON code Toolbar which flags opened pages that have valid HON code certification.^25^ The HON code is a series of eight voluntary principles for websites containing health information. Certification demonstrates the page meets a minimum standard for the ethical presentation of information and informing readers about the source and purpose of the information they are reading.^26^

Each page was assessed against the JAMA benchmark criteria by two reviewers who determined if each criterion was met via consensus. The four criteria are designed to assess whether material is authentic and reliable. These criteria are: authorship, are the authors, their affiliations and credentials listed; attribution, are references for all sources and copywrites listed; disclosure, is site ownership, sponsorship, and funding clearly displayed; and currency, are the dates content was posted and uploaded visible.^27^ JAMA benchmark criteria have previously been assessed individually, or as the sum out of four.^28^, ^29^

The DISCERN quality criteria for consumer health information is a standardized index, validated for assessing the quality of patient education materials.^30^ It was designed to be performed without any specialist knowledge on the topic under assessment. It involves 16 questions within three sections—questions 1 to 8 cover publication reliability and trustworthiness, questions 9 to 15 cover treatment choices, and question 16 covers overall quality—using a 5‐point Likert‐type scale, for a total score ranging from 16 to 80^30^ (see Table S1). Two researchers independently assessed pages using the DISCERN instrument. Both were trained on the DISCERN tool and undertook sample data collection on 20 pages on a different topic to reduce variance in the reviewer's interpretation of the results.

### 2.5 Statistical analysis

All normally distributed data were reported with mean, standard deviation, and 95% confidence intervals. All non‐normally distributed data were reported with median and interquartile range. Kendall's tau (*τ*) was used to assess for rank‐order correlation between: readability scores and Google rank; readability scores and DISCERN scores; and DISCERN scores and readability scores. Mann‐Whitney U and independent samples *t* tests were used to compare DISCERN scores and readability scores depending on HON code certification. A Kruskal‐Wallis test with post hoc Mann‐Whitney U test was used to compare DISCERN scores between different types of webpage source. A linear weighted kappa (*κ*) was used to determine the inter‐rater reliability of reviewers using the DISCERN instrument. A multiple variant regression analysis was conducted using JAMA benchmark criteria to predict total DISCERN score, using each of the JAMA benchmark criterion.

## 3 RESULTS

A total of 81 webpages were included after the exclusion criteria were applied; 13 were excluded as scientific publications, 4 were excluded for not containing written information on ED, and 2 were excluded for being behind paywalls. The most common sources of webpage were urologists and hospitals that produced 15 each (19%), while 14 (17%) were commercial and attempting to sell a product. A total of 15 (19%) pages included advertisements. A total of 74 (91%) pages included specific information about one or more treatment options for ED (see Table 1). The most common treatment discussed was phosphodiesterase type‐5 inhibitors (PDE5I) with information found on 55 pages (68%). The least frequently listed treatments were shockwave therapy, described on four (6.2%) pages, and platelet‐rich plasma injections, described on one (1.2%) page.

**TABLE 1:** Described treatments for erectile dysfunction

Treatment	n (%)
Phosphodiesterase Type‐5 inhibitors	55 (68%)
Lifestyle modification	39 (48%)
Vacuum‐assisted devices	38 (47%)
Intracavernosal injections	34 (42%)
Penile prosthesis surgery	34 (42%)
Psychotherapy	26 (32%)
MUSE suppositories	21 (26%)
Testosterone supplementation	19 (24%)
Herbal/over counter medicine	11 (14%)
Revascularization surgery	7 (8.6%)
Shock wave therapy	4 (4.9%)
Platelet‐rich plasma injections	1 (1.2%)

Seventeen (21%) of the included webpages were HON code certified. The median JAMA benchmark score was 1 (IQR 1‐2) out of 4, with 17 (21%) meeting authorship criteria, 18 (22%) meeting attribution criteria, 32 (4.0%) meeting currency criteria, and 76 (94%) meeting disclosure criteria.

Strong and very strong positive correlations were observed between all readability measures (see Figure S1). Taken together with the results of Horn's parallel analysis and a Scree Plot, both of which suggest that the measures captured a single concept, this supported their summation into a combined readability score on which the webpages had a mean of 12.3 (SD 1.91), requiring education equivalent to grade 12 for comprehension.

Inter‐rater reliability in DISCERN scores was strong with *κ* of 0.85. The median overall DISCERN score was 35 (IQR 26‐44.5) out of 80. The median score for each DISCERN subsection was: 16 (IQR 12‐21.25) out of 40 for subsection one, 16 (IQR 12‐20.5) out of 35 for subsection two, and 2 (IQR 1‐3) out of 5 for subsection three. There were large positive correlations among all DISCERN subsections and with the overall DISCERN score (see Table 2).

**TABLE 2:** Kendall's *τ* correlations between DISCERN scores

	Subsection 1	Subsection 2	Subsection 3
Subsection 1	1		
Subsection 2	*τ* = 0.515, *P* < .001	1	
Subsection 3	*τ* = 0.625, *P* < .001	*τ* = 0.807, *P* < .001	1
Overall	*τ* = 0.775, *P* < .001	*τ* = 0.755, *P* < .001	*τ* = 0.794, *P* < .001

**FIGURE 1:**
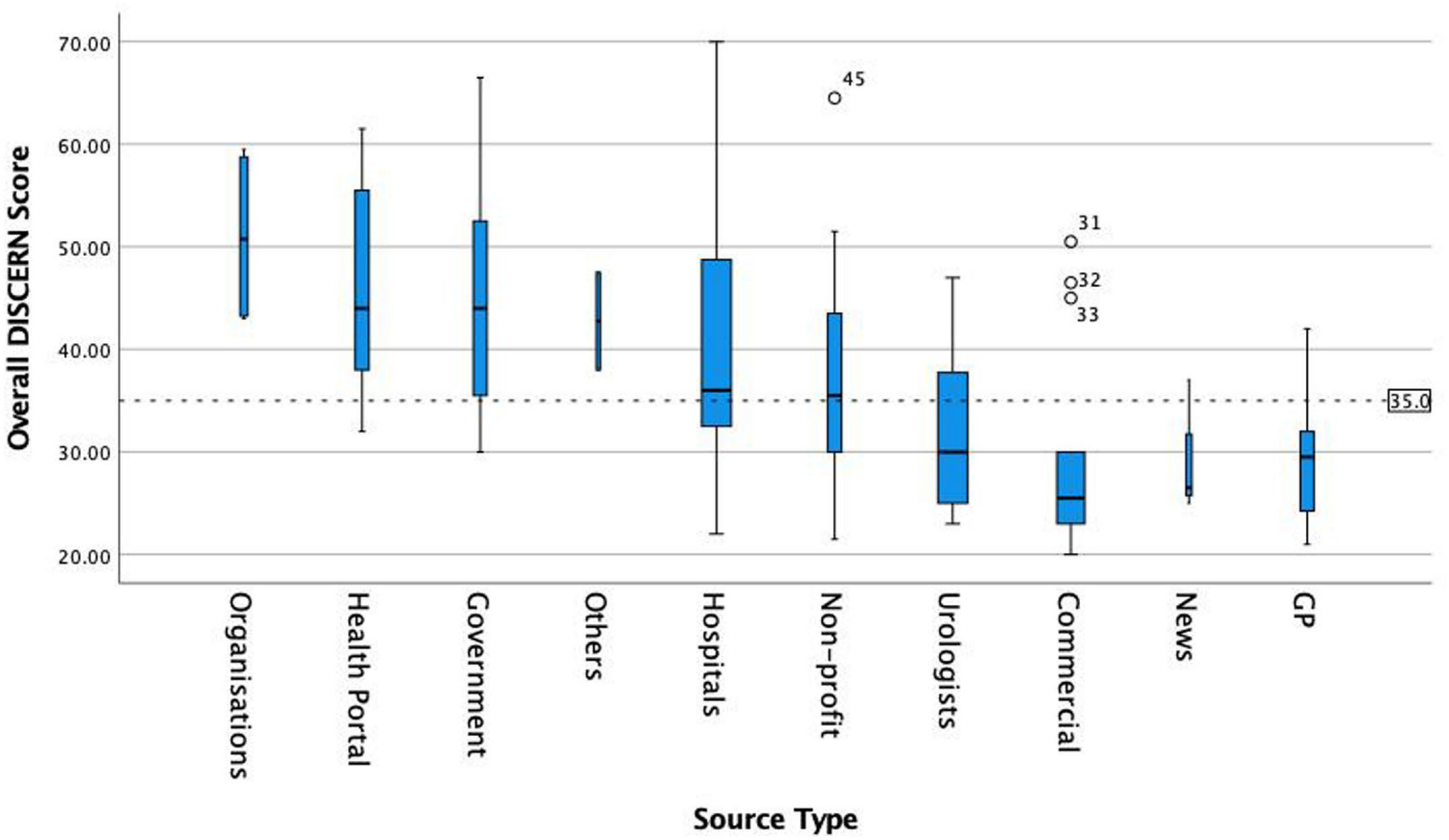
Boxplot of overall DISCERN score grouped by source type. Width of boxes and error bars scaled to source type frequency. Grand median ^34^ represented by dashed line

When comparing overall DISCERN score between producers (see Figure 1 and Table S2) those produced by organizations had the highest median score of 50.75 (IQR 43.25‐58.75), while those trying to sell a product (commercial) had the lowest median score of 25.5 (IQR 23‐30). A Kruskal‐Wallis H test found a significant difference in DISCERN scores depending on website production type H(9) = 25.6, *P* = .002. There were, however, no significant differences in overall DISCERN score between the groups in post hoc pairwise Mann‐Witney U test when Bonferroni corrections for multiple comparisons were applied.

There was a small yet statistically significant negative correlation between overall DISCERN score and Google rank, *τ* = −0.160, *P* = .036. This pattern, where those that appear further down in Google search results were significantly more likely to have lower quality scores, was also observed for subsection one DISCERN scores, *τ* = −0.222, *P* = .004. There was, however, no significant correlation between Google rank and subsection two or Google rank and subsection three DISCERN scores (*τ* = −0.0854, *P* = .27, and *τ* = −0.0875, *P* = .29, respectively).

There was no significant correlation between any DISCERN scores and the combined readability score (see Table 3).

**TABLE 3:** Kendall's *τ* correlations between combined readability and DISCERN scores

Overall DISCERN	DISERN Section 1	DISCERN Section 2	DISCERN Section 3
*τ* = −0.089	*τ* = −0.105	*τ* = −0.075	*τ* = −0.122
*P* = .243	*P* = .171	*P* = .325	*P* = .138

HON code certified webpages had significantly higher overall DISCERN scores with a median of 44 (IQR 35‐58.5) vs 32.5 (IQR 25.25‐42.25), Mann‐Whitney U = 833, *Z* = 6561, *P* < .001. In contrast, there were no significant differences between average scores on the combined readability measure for HON code certified webpages ( $\bar{x}$ = 12.0, SD = 1.38) and non‐certified webpages ( $\bar{x}$ = 12.4, SD = 2.04), *t*(36.9) = 0.85, *P* = .4; scores for both groups approximated normal distributions with no evidence to reject the assumption of homogeneity of variances from a non‐significant Levene's test, F(1, 79) = 2.36, *P* = .1.

A linear regression was used to predict overall DISCERN scores from the JAMA benchmark criteria; it explained a significant proportion of the variance, *R^2^* = 0.368 (adjusted *R^2^* = 0.352), F(2, 78) = 22.7, *P* < .001. Within this regression model, the effects of meeting the criterion for attribution and currency was significant; both resulted in small positive increases in overall DISCERN scores holding constant the effects of meeting the other criteria (*β* = 11.09, 95% CI [5.22‐16.95], standardized *β* = 0.37 and *β* = 8.79, 95% CI [3.80‐13.77], standardized *β* = 0.35, respectively). The criterion authorship and disclosure were non‐significant (*P* = .4 and *P* = .9) with standardized *β* if included −0.09 and 0.01, respectively.

## 4 DISCUSSION

While many people use the internet to search for information on ED, little is known about the quality of this information and whether it is prone to commercial biases or written above the readability of the target audience, as has been observed in popular webpages on other medical topics. To the best of our knowledge, this study is only the second to assess the quality of information on the topic of ED and is the first to review written information that specifically focuses on treatment. The majority of webpages found in the search for ED treatment do include specific information on treatment options, which is more than the 67% of YouTube videos reviewed by Fode et al.^17^ While this may be related to a narrowing of the search by including the term “treatment” in this study, the high incidence of treatment option discussions is notable. If patients interested, in improving their symptoms of ED, are to obtain any use from webpages, it is necessary at the bare minimum they contain this information.

All treatments for ED recommended in the AUA and European Association of Urology guidelines were discussed among Google search results, however, no pages contained information on all treatments.^31^, ^32^ It is not surprising that PDE5I therapy was discussed most frequently given it is used as first‐line pharmacotherapy, and in some countries such as the United Kingdom is available without prescription.^33^ Although descriptions of herbal or over the counter medicines and platelet‐rich plasma injections were infrequent, health professionals must be aware of their limited efficacy and experimental nature to accurately counsel patients who may find this material online.^31^, ^34^

Unfortunately, the overall quality of webpages assessed in this study was poor, reflected in the median DISCERN score of 35 out of 80; an average of 2.2 out of 5 for each DISCERN question. This is consistent with many studies reviewing the quality of online resources on urological and other medical topics.^15^ Given the potential for a bias in trying to increase sales, it is unsurprising that commercial type webpages were the lowest quality. However, general practitioners and urologists had lower median scores. This is concerning, as these medical specialties commonly treat ED.^35^ Health professionals, particularly urologists and general practitioners should review their online publications using DISCERN prior to publication to improve quality for patients.

There were, however, a minority of high‐quality resources, with three webpages scoring more than 64 out of 80 (above an average of 4 per question) overall DISCERN score. Helping to better identify these resources for patients is essential for improving the utility of online searches for medical information.

In this study, HON code certification was a significant predictor of higher quality resources as assessed by the DISCERN instrument; they scored a median of 11.5 out of 80 higher than those without. Although this has not always been found in previous studies on other medical topics,^36^, ^37^, ^38^ HON code certification offers an easy marker of resource quality that may assist patients to identity quality resources on ED treatment. This can be assisted with tools like the HON code toolbar; a web‐browser plugin that compares websites against a database of HON certified websites, providing notification of all sites that are certified. Such labeling may be particularly useful for patients with lower levels of health literacy. To optimize this all clinicians, particularly those involved in primary care, would need to promote such tools to patients to encourage uptake.

On average, this study found a senior high school reading level is required to interpret webpages on ED treatment, which is consistent with previous studies on other urological and other medical conditions.^14^, ^39^, ^40^ This is much higher than the USA recommended fifth‐grade reading level, and the sixth‐ to eighth‐grade level recommended by most Australian state health departments.^10^ This may limit resource utility for many patients, especially when the incidence of poor health literacy and ED both increase with aging.^2^, ^7^ To address these clinicians involved in the online publication on information on ED treatment, they must aim to use simple language.

Whether or not a webpage on ED treatment meet each of the JAMA benchmark criterion for attribution and currency was found to explain 37% of the variation in DISCERN scores. This highlights the importance of these criterion as markers for identifying quality resources. However, the notable unexplained variance suggests other untested variables affect webpage quality.

This study is not without limitations. Assessing readability using a numeric measure related to word count and sentence length, may not take into account other aspects of a resource that may contribute to patient understanding. These include but are not limited to: embedded videos, charts, diagrams, and photographs. Future studies should assess these features using existing instruments like transcribing dialog from videos to text to assess with methods used in this study, or use novel instruments specifically tailored to that medium.

The DISCERN instrument does not measure the validity of included information, rather it measures completeness of information. While a well‐presented resource with incorrect information could score highly, the referencing material required to score well would highlight its poor veracity. While coding data during this study no webpages, that were well referenced, were found to have clear erroneous material. Despite this, those using the DISCERN criteria to assess material should remember to check references and sources to ensure the quality of a resource.

This study was conducted from an Australian IP address, therefore, the results returned were tailored to the location from which the search was conducted (Brisbane, Queensland, Australia). While steps were taken to minimize the role prior search history played using a clean installation of Google Chrome, these factors will have affected the results presented. In future this could be mitigated by conducting multiple searches from different locations to obtain non‐geographically biased results.

## 5 CONCLUSION

This study found the quality of information on ED treatment obtained via a google search was generally poor. However, certain webpages were of a higher standard, and can be identified by markers of certification such as HON, as well as easier to assess criteria like JAMA benchmarks for attribution and currency. Strategies to improve patient's access to quality information on ED treatment should include improving use of simple language, improving patient education regarding markers of quality information, and implementing better standards for information presented online by medical professionals.

## CONFLICT OF INTEREST

The following disclosure statements have been prepared using the ICMJE Form for Disclosure of Potential Conflict of Interest. Dr Pattenden has nothing to disclose. Ms Raleigh has nothing to disclose. Ms Pattenden has nothing to disclose. Dr Thangasamy reports grants from Movember Prostate Cancer Quality Improvement Grant Recipient, outside the submitted work.

## Supporting information

Fig S1

Table S1

Table S2
