# Supplementary figures and images for: Quality and readability of online patient information on treatment for erectile dysfunction
Source: BJUI Compass. 2021 May 6;2(6):412–8. doi: 10.1002/bco2.87 (PMC8988690; doi:10.1002/bco2.87)

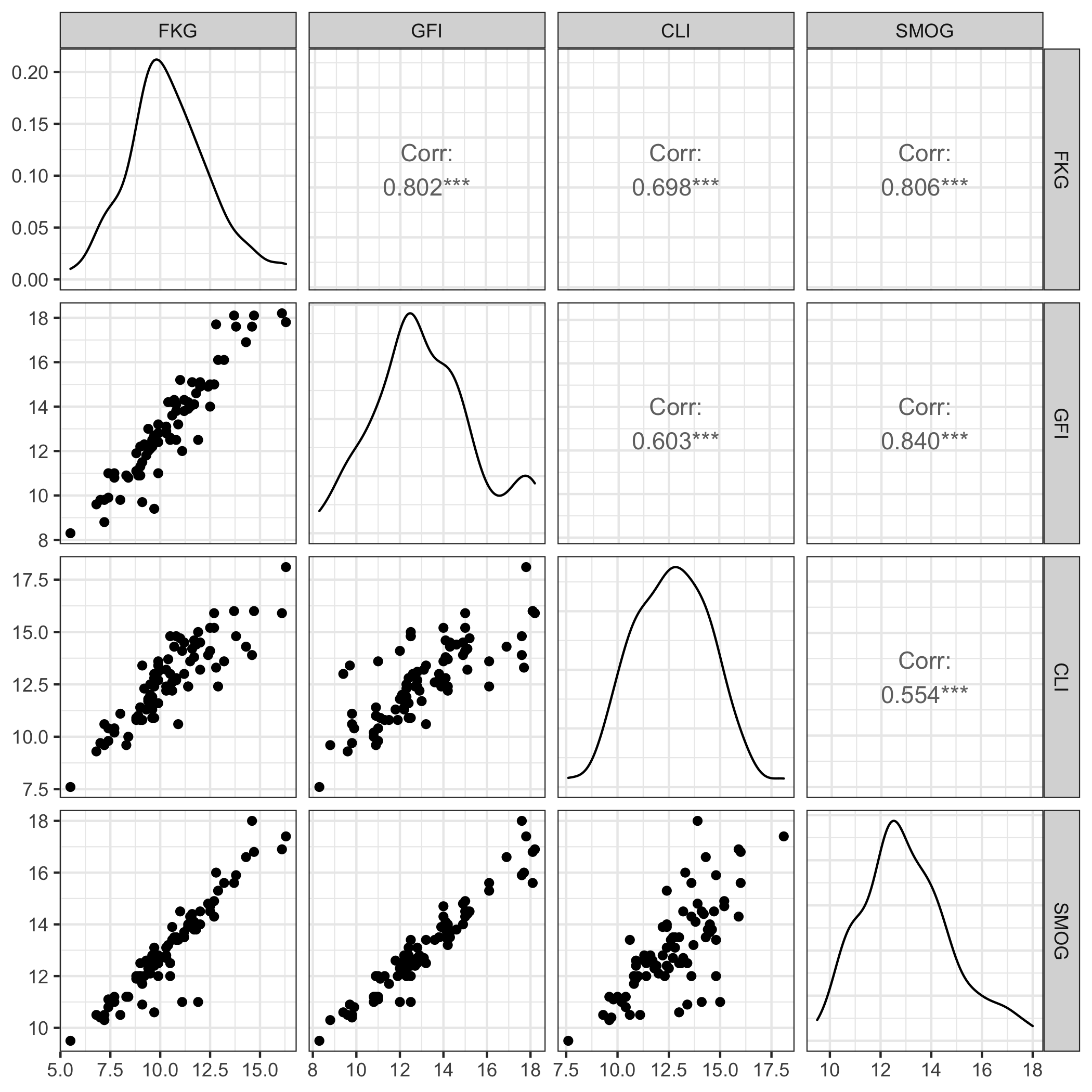

Supplement: Supplementary file 1 — Fig S1 [file BCO2-2-412-s003.png]
