# Supplementary material for: Quality and readability of online patient information on treatment for erectile dysfunction
Source: BJUI Compass. 2021 May 6;2(6):412–8. doi: 10.1002/bco2.87 (PMC8988690; doi:10.1002/bco2.87)
Supplement: Supplementary file 2 — Table S1 [file BCO2-2-412-s001.docx]

| Table S1: The DISCERN Instrument (31) | |
| --- | --- |
| Section 1: Is the publication reliable? | |
| 1 | Are the aims clear? |
| 2 | Does it achieve its aims? |
| 3 | Is it relevant? |
| 4 | Is it clear what sources of information were used to compile the publication (other than the author or producer)? |
| 5 | Is it clear when the information used or reported in the publication was produced? |
| 6 | Is it balanced and unbiased? |
| 7 | Does it provide details of additional sources of support and information? |
| 8 | Does it refer to areas of uncertainty? |
| Section 2: How good is the quality of information on treatment choices? | |
| 9 | Does it describe how each treatment works? |
| 10 | Does it describe the benefits of each treatment? |
| 11 | Does it describe the risks of each treatment? |
| 12 | Does it describe what would happen if no treatment is used? |
| 13 | Does it describe how the treatment choices affect overall quality of life? |
| 14 | Is it clear that there may be more than one possible treatment choice? |
| 15 | Does it provide support for shared decision-making? |
| Section 3: Overall Ratings of the Publication | |
| 16 | Based on the answers to all of the above questions, rate the overall quality of the publication as a source of information about treatment choices |
