# Supplementary material for: Quality and readability of online patient information on treatment for erectile dysfunction
Source: BJUI Compass. 2021 May 6;2(6):412–8. doi: 10.1002/bco2.87 (PMC8988690; doi:10.1002/bco2.87)
Supplement: Supplementary file 3 — Table S2 [file BCO2-2-412-s002.docx]

| Table S2: Overall DISCERN Score By Producer Type | | |
| --- | --- | --- |
| Producer Type | Median | Interquartile Range |
| Commercial | 25.5 | 23 – 30 |
| Government | 44 | 25.5 – 52.5 |
| Health Portal | 44 | 38 – 55.5 |
| News | 26.5 | 25.75 – 31.75 |
| Non-Profit | 35.5 | 30 – 43.5 |
| Others | 42.75 | 38 – 47.5 |
| General Practitioners | 29.5 | 24.25 – 32 |
| Urologists | 30 | 25 – 37.75 |
| Hospitals | 36 | 32.5 – 48.75 |
| Organisations | 50.75 | 43.25 – 58.75 |
